# FDG PET/CT Depicting Right Iliac Vein Tumor Thrombosis following Low Anterior Resection in Rectal Cancer Patient: A Case Report and Literature Review

**DOI:** 10.1055/s-0043-1771288

**Published:** 2023-09-06

**Authors:** Akram Al-Ibraheem, Serin Moghrabi

**Affiliations:** 1 Department of Nuclear Medicine, King Hussein Cancer Center, Amman, Jordan

**Keywords:** rectal cancer, tumor thrombus, FDG PET/CT, nuclear medicine

## Abstract

Venous tumor thrombus is a rare complication of rectal cancer but is more common in other types of cancer, like renal cell carcinoma and hepatocellular carcinoma. The usual site of tumor thrombus in rectal cancer patients is the inferior mesenteric vein (IMV), which is seldom seen in the common iliac vein, with only a few cases reported till now. We present a case of fluorodeoxyglucose (FDG) avid right iliac vein tumor thrombosis after low anterior resection in a patient with rectal cancer and review the literature.

## Introduction

Venous tumor thrombi are rare in colorectal cancer, found in only 1 to 2% of patients. 1 They usually appear in the inferior mesenteric vein (IMV) in patients with rectal cancer and are seldom seen in the common iliac vein. 2 Inflammatory infiltrates play a crucial role in venous thrombus development, which can be detected at an early stage with high sensitivity and specificity using fluorodeoxyglucose positron emission tomography computed tomography (FDG-PET/CT) through focally increased FDG uptake. 3 4 This study describes a case of a rare venous tumor thrombus in the iliac vein following radical resection of a rectal tumor.

## Case Report

A 61-year-old woman presented with a 6-month history of progressive constipation, anal pain, and weight loss. The clinical examination was unremarkable. On blood workup, complete blood counts showed a high carcinoembryonic antigen of 16.4 ng/mL. Colonoscopy showed a mass causing significant stenosis. The diagnosis was made based on histopathology of the biopsy, which confirmed the diagnosis of invasive moderately differentiated adenocarcinoma.

Magnetic resonance imaging (MRI) showed that the circumferential lesion was located in the lower part of the rectum, with peritoneal and cervix invasion and numerous mesorectal lymph nodes and tumor deposits. The preoperative clinical stage was T4N2b.

After completion of 5 weeks of capecitabine and 25 fractions of external beam radiation therapy (EBRT), CT, and MRI showed two new right lobe hepatic lesions, with partial regression of the rectal mass. After this, four cycles of FOLFOX followed by FOLFIRI and bevacizumab were given to the patient before performing a surgical intervention, including laparoscopic-assisted low anterior resection, right hepatectomy, diverting colostomy, and total abdominal hysterectomy. Pathologic examination after surgery revealed a stage IIIA, moderately differentiated, KRAS mutated adenocarcinoma with cervix invasion and liver metastases. Immunohistochemical stains for mismatch repair (MMR) proteins showed intact nuclear expression for PMS2, MLH1, MSH6, and MSH2. The circumferential resection margin (CRM) was negative (6 mm).

**Fig. 1:**
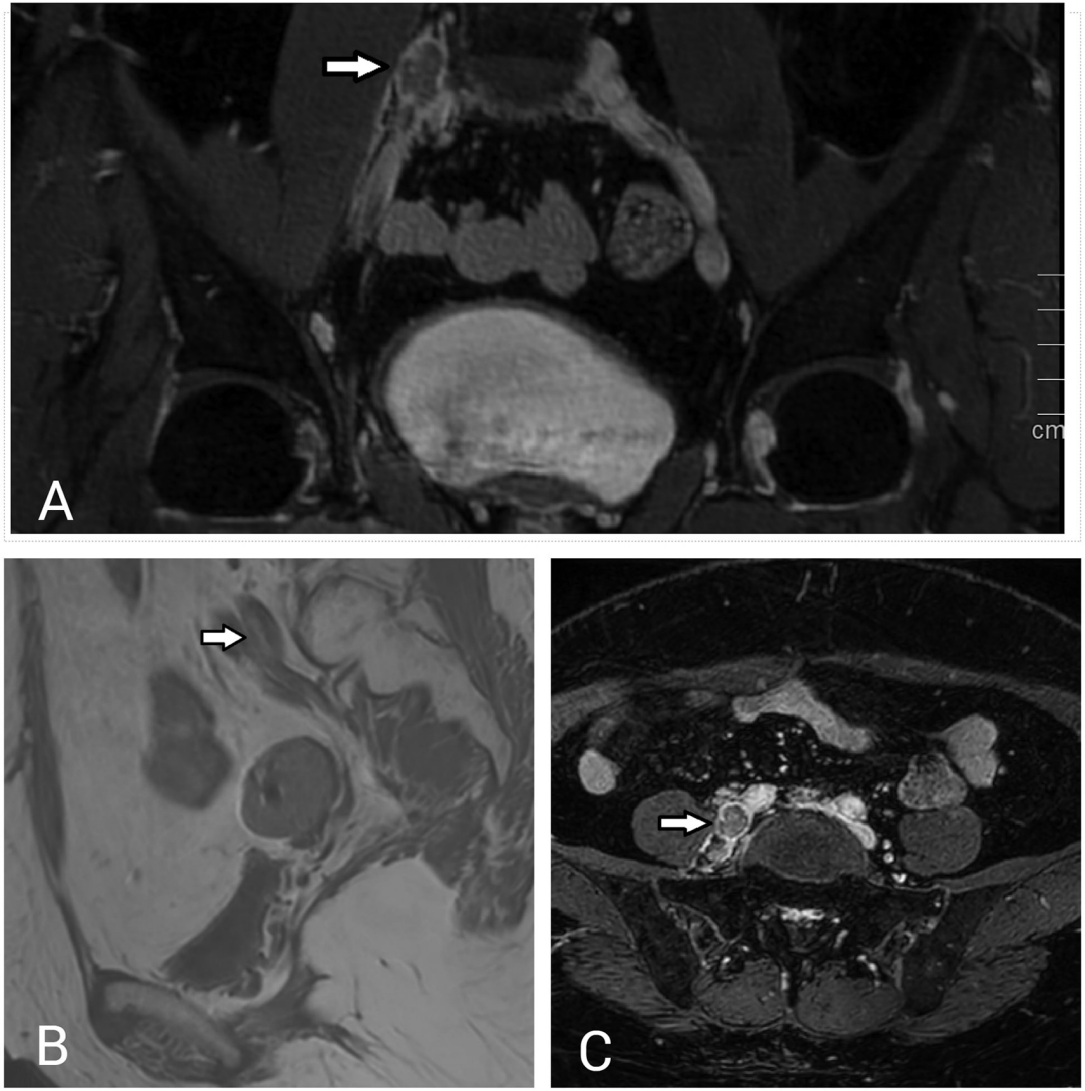
( **A** , **B** ) Magnetic resonance imaging (MRI) coronal balanced steady-state free precession and sagittal T1-weighted views demonstrate disseminated thrombus in the right common iliac vein. Axial view of balanced steady-state free precession ( **C** ) demonstrates thrombus in the right common iliac vein and hypermetabolic lymph node at the inferior aorta bifurcation region.

After this, the patient presented with right lower limb deep vein thrombosis (DVT) and right upper limb weakness, which were treated with rivaroxaban 40 mg × 2. A follow-up CT showed hypoattenuating lesions inferior to the abdominal aortic bifurcation. This required correlation with MRI, revealing a few enlarged necrotic bilateral common iliac lymph nodes and an elongated segmental right iliac vein thrombus ( Fig. 1 ). The patient's serum carcinoembryonic antigen was 4.9 ng/mL; other parameters were normal.

**Fig. 2:**
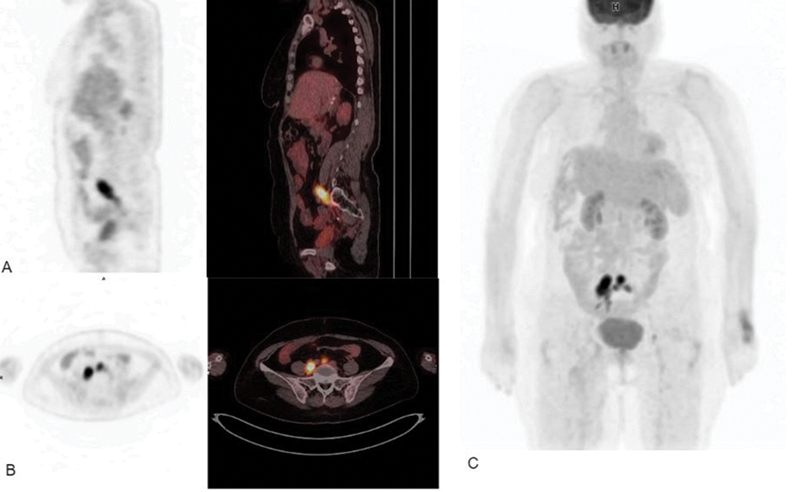
( **A** ) Positron emission tomography/computed tomography (PET/CT) sagittal view demonstrates intensely hypermetabolic malignant disseminated thrombus in the right common iliac vein. ( **B** ) PET and PET/CT axial view images demonstrate an intensely hypermetabolic malignant thrombus in the right common iliac vein, and hypermetabolic lymph node at the inferior aorta bifurcation region. ( **C** ) Maximum intensity projection also shows the disseminated tumor thrombus of the right iliac vein and the regional hypermetabolic lymph nodes. Physiological fluorodeoxyglucose (FDG) uptake is seen in the brain, kidneys, and urinary bladder.

FDG PET/CT scan was requested for the patient, because of its high diagnostic efficacy in determining disease recurrence, assessing the presence of metastasis, and detecting tumor thrombus by discerning the high glucose metabolic activity of malignant cells and providing the metabolic information of tumor thrombus. In this study, an FDG PET/CT scan confirmed the findings of few hypermetabolic common iliac lymph nodes as well as an intensely hypermetabolic elongated disseminated right iliac vein thrombus with maximum standardized uptake value (SUVmax) of 28, which led to the diagnosis of a malignant tumor thrombus rather than a benign venous thrombus due to the intensity of FDG uptake by the thrombus ( Fig. 2 ).

## Discussion

A variety of prognostic factors play a role in the prognosis for colorectal cancer, including the histotype, differentiation, extension of the primary tumor, and the presence of local and/or distant metastases. The latter is the most important prognostic factor in determining treatment type. 5

Tumor thrombosis is primarily found in patients with liver cancer, renal carcinoma, and adrenal tumors, but is a rare complication of rectal cancer patients. However, a recent study found that moderately differentiated adenocarcinoma has the potential to develop venous tumor thrombosis, 6 which may develop in the IMV, inferior vena cava (IVC), and internal iliac vein. 7 Because the distal rectum has double venous drainage: to the portal system via the IMV, which leads to a metastatic spread to the liver, and the IVC via the pelvic veins. 8

FDG PET/CT is a highly effective imaging technique that can provide more precise and timely prognostic indicators of chemotherapy response compared to the conventional Response Evaluation Criteria in Solid Tumors (RECIST) criteria because FDG PET/CT can provide the metabolic information of the tumor thrombus based on the high glucose metabolic activity of malignant cells, which helps in distinguishing between malignant and benign lesions. Moreover, FDG PET/CT plays a role in detecting active malignant thrombosis, differentiating it from bland thrombus, by discerning the high glucose metabolic activity of malignant cells and providing the metabolic information of the tumor thrombus, as studies have shown that FDG PET/CT can detect and monitor early thrombosis with high accuracy, significantly reducing associated morbidity and mortality. 5 9

Several studies and case reports have demonstrated the efficacy of FDG PET/CT in diagnosing tumor thrombus and establishing a clear cutoff between tumor and benign thrombi. For example, Al-Ibraheem et al reported the formation of an FDG-avid tumor thrombus in a 70-year-old renal cell carcinoma (RCC) patient due to mass infiltration through the lumbosacral plexus and vessels into the pelvis and the internal iliac vein. 10 Similarly, Jain et al also reported the discovery of an FDG avid isolated malignant portal vein tumor thrombus in sigmoid colon cancer. 11 Erhamamci et al found that FDG PET/CT can distinguish between tumor thrombus and venous thromboembolism by detecting significantly higher FDG uptake by tumor thrombus. This distinction has no cutoff point. 12 On the other hand, Hu et al considered the SUVmax measurement with a threshold value of 3.36 as a useful tool for differentiating between tumor and venous thrombi. 13

Because of the rarity of tumor thrombus after rectal cancer surgery, especially when it is located in the common iliac vein, this case is of particular significance. In such cases, the gold standard in the diagnosis of tumor thrombi is conventional venography, 14 but FDG PET/CT can help differentiate between venous thrombi and tumor thrombi hinging on the high glucose metabolic activity of malignant cells, 15 which should be performed before interventional angiography is conducted.

## Conclusion

Our case report emphasizes the importance of physicians being aware of such a presentation when rectal cancer patients experience swelling and weakness in their legs following surgery. To avoid further complications, the physician should use FDG PET/CT to rule out tumor thrombus as a possible cause and treat accordingly.
